# Mild renal insufficiency and attributable risk of adverse In-hospital outcomes in patients with Acute Coronary Syndrome from the improving care for Cardiovascular Disease in China (CCC) project

**DOI:** 10.1186/s12882-022-02663-4

**Published:** 2022-01-13

**Authors:** Fengbo Xu, Guoqin Wang, Nan Ye, Weijing Bian, Lijiao Yang, Changsheng Ma, Dong Zhao, Jing Liu, Yongchen Hao, Jun Liu, Na Yang, Hong Cheng

**Affiliations:** 1 Department of Nephrology, Beijing Anzhen Hospital, Capital Medical University, No. 2 Anzhen Rd, District, 100029 Beijing, China; 2 Department of Cardiology, Beijing AnZhen Hospital, Capital Medical University, Beijing, China; 3 Deparment of Epidemiology, Beijing AnZhen Hospital, Beijing Institute of Heart, Lung and Blood Vessel Diseases, Capital Medical University, Beijing, China

**Keywords:** Acute coronary syndrome, Mild renal insufficiency, Attributable risk, MACEs

## Abstract

**Background:**

Renal insufficiency (RI) is a frequent comorbidity among patients with acute coronary syndrome (ACS). We aimed to evaluate the attributable risk associated with mild RI for the in-hospital outcomes in patients with ACS.

**Methods:**

The Improving Care for Cardiovascular Disease in China-ACS (CCC-ACS) Project was a collaborative study of the American Heart Association and the Chinese Society of Cardiology. A total of 92,509 inpatients with a discharge diagnosis of ACS were included. The attributable risk was calculated to investigate the effect of mild RI (eGFR 60-89 ml / min · 1.73 m^2^) on major adverse cardiovascular events (MACEs) during hospitalization.

**Results:**

The average age of these ACS patients was 63 years, and 73.9% were men. The proportion of patients with mild RI was 36.17%. After adjusting for other possible risk factors, mild RI was still an independent risk factor for MACEs in ACS patients. In the ACS patients, the attributable risk of eGFR 60-89ml/min·1.73m^2^ to MACEs was 7.78%, 4.69% of eGFR 45-59 ml/min·1.73m^2^, 4.46% of eGFR 30-44 ml/min·1.73m^2^, and 3.36% of eGFR<30 ml/min·1.73m^2^.

**Conclusion:**

Compared with moderate to severe RI, mild RI has higher attributable risk to MACEs during hospitalization in Chinese ACS population.

**Supplementary Information:**

The online version contains supplementary material available at 10.1186/s12882-022-02663-4.

## Background

In 2018, an estimated 11 million Chinese were affected by coronary heart disease (CHD) [1]. The aging population and increasing prevalence of cardiovascular disease risk factors [2, 3] will lead to a growing burden of CHD [4, 5]. The population affected by CHD is predicted to increase to 22.6 million by 2030 [6]. The mortality rate of CHD in China reached 1.39 million in 2013 [7].

It is generally believed that hypertension, diabetes, smoking and hyperlipidemia are the risk factors for CHD [8–10]. In addition, renal insufficiency (RI) is also one of the important risk factors for CHD [11]. Many studies had shown that in the patients with CHD, RI was an independent predictor for short- and long-term prognosis [12–14]. Moreover, studies have confirmed that even mild RI increases the risk of adverse outcomes during hospitalization[15–18].But the information about the attributable risk associated to mild RI in patients with ACS for MACEs is scarce too. Therefore, the purpose of this study is not only to evaluate the impact of mild RI on hospitalization outcomes, but also to analyze the attributable risk of mild RI on adverse outcomes during hospitalization.

## Methods

### Research design

Details of the design and methodology of the CCC-ACS project have been published [19]. Briefly, it is a national, hospital-based quality improvement project with an ongoing database, aiming to increase adherence to ACS guidelines in China and to improve patient outcomes. It was launched in 2014 as a collaborative initiative of the American Heart Association (AHA) and Chinese Society of Cardiology (CSC). 240 hospitals were recruited representing the diversity of ACS care in hospitals in China, including 160 tertiary hospitals and 80 secondary hospitals. Clinical data were collected via a web-based data collection platform (Oracle Clinical Remote Data Capture, Oracle Corporation). Trained data abstractors entered the data elements abstracted from medical charts. Eligible patients were consecutively reported to the CCC-ACS database for each month before the middle of the following month. Around 5% of reported cases were randomly selected and compared with the original medical records. An audit by a third party was performed to ensure that cases were reported consecutively rather than selectively. This research has been registered in https://clinicaltrials.gov (NCT02306616). All methods were performed in accordance with the relevant guidelines and regulations.

### Research population

A total of 92,509 inpatients with ACS, identified based on their principal diagnosis at discharge, were enrolled from 240 hospitals across China from November 2014 to December 2019. Based on the eGFR, all the patients were further divided into ≥90 ml/min·1.73m^2^ group, 60-89 ml/min·1.73m^2^ group, 45-59 ml/min 1.73 m^2^ group, 30-44 ml/min 1.73 m^2^ group and < 30 ml/min 1.73 m^2^ group. Institutional review board approval was granted for the aggregate data set for research and quality improvement by the Ethics Committee of Beijing Anzhen Hospital, Capital Medical University. No informed consent was required.

### Difinition of mild RI

Mild RI is defined as eGFR 60-89 ml / min · 1.73m^2^.

### Definition of in-hospital outcomes

Major adverse cardiovascular events (MACEs) were defined as a combination of death, heart failure, cardiac arrest, and cardiac shock.

### Definition of other Variables

The ACS classification was based on the primary diagnosis at discharge in the medical record. Non-ST-segment elevation ACS was defined as non-ST-segment elevation myocardial infarction (STEMI) or unstable angina. Hypertension was defined as having a history of hypertension, receiving antihypertensive therapy, or having a systolic blood pressure≥140mmHg or diastolic blood pressure≥90mmHg at admission. Diabetes mellitus was defined as having a previous or new diagnosis of diabetes mellitus, receiving oral hypoglycemic drug therapy or insulin therapy, or having a HBA1C≥6.5%. Hyperlipidemia was defined as having a history of hyperlipidemia, receiving lipid-lowering drugs, or having a serum LDL-C≥1.8mmol/L at admission. Current smoking was defined as smoking in the preceding 1 year according to the medical records of the patients.

Creatinine was collected on the day of admission.The baseline eGFR was calculated using the Modification of Diet in Renal Diseases (MDRD) equation for Chinese patients: eGFR (mL/min๒1.73m^2^)=175 x SCr (mg/dl)^-1.234^ x Age^-0.179^ (x 0.79 for women) [20].

All the laboratory testing values were the values tested the first time after admission.

### Statistical methods

Continuous variables with normal distribution were presented as mean±standard deviation, and ANOVA analysis was used for univariate comparison. On the other hand, those with non-normal distribution were represented as median and interquartile range, and Wilcoxon-Mann-Whitney test was used for univariate comparison. The categorical variables were reported as number of cases and percentages, and the chi-square test was used for univariate comparison. A multivariate logistic regression model was used to determine the association between the eGFR and in-hospital outcomes by controlling for potential confounders. Candidate adjustment factors included age, history of hypertension, diabetes mellitus, heart failure, atrial fibrillation, stroke, previous PCI or CABG, ACS type, heart failure at admission, cardiogenic shock at admission, cardiac arrest at admission, Killip class at admission, systolic pressure at admission, taking antiplatelet drugs before admission, taking β-blocker before admission and taking ACEI/ARB before admission, HB at admission, LDL-C at admission. The attributable risk (AR)[21, 22] was calculated to investigate the effect of mild RI on MACEs during hospitalization.

$$\mathrm{AR}=\frac{{\displaystyle\sum_{i\mathit=\mathit0}^k}pi\times\left(OR_i-1\right)}{1+{\displaystyle\sum_{i=0}^k}pi\times\left(OR_i-1\right)}\times100$$

The subscript **i** denotes each exposure level; *p*_i_ is the proportion of the study population in the exposure level i, and OR is the odds ratio for the exposure level i compared with the unexposed (reference) level.

For data with missing values lower than 15% (Additional file:Table S2), the sequential regression multiple imputation method implemented by IVEware software version 0.2 (Survey Research Center, University of Michigan, Ann Arbor, MI, USA) was used to imputed the missing values.

SPSS software version 22.0 (IBM Inc, Armonk, NY, USA) was used to analyze the data. For all analyses, P<0.05 was considered as statistically significant.

## Results

### Patients’ characteristics

This study included 92,509 ACS patients from 240 hospitals across China. The clinical characteristics of these patients are summarized in Table 1. The average age of these ACS patients was 63 years, and 73.9% of them were men.

**Table 1:** Baseline characteristics of ACS patients

	Total (*n*=92,509)	eGFR≥90ml/min·1.73m^2^ (*n*=42,508, 45.95%)	eGFR60-89 ml/min·1.73m^2^ (*n*=33,462, 36.17%)	eGFR45-59 ml/min·1.73m^2^ (*n*=8383, 9.06%)	eGFR30-44 ml/min·1.73m^2^ (*n*=4980, 5.38%)	eGFR< 30 ml/min·1.73m^2^ (*n*=3176, 3.43%)		*P* value
Male gender	68,370(73.9)	34,036(80.1)	23,995(71.7)	5462(65.2)	3005(60.3)	1872(64.1)	<0.001	
Age(years)	63.21±12.45	59.57±10.36	67.46±11.01	71.20±10.85	72.47±11.39	71.50±11.74	<0.001	
ACS type								
STEMI	55,574(60.1)	27,154 (63.9)	19,425(58.1)	4809(57.4)	2667(53.6)	1519(47.8)	<0.001	
NSTE-ACS	36,933(39.9)	15,352(36.1)	14.37(41.9)	2574(42.6)	2313(46.4)	1657(52.2)		
Previous MI	7561(8.2)	2743(6.5)	2896 (8.7)	883(10.5)	591(11.9)	448(14.1)	<0.001	
Previous PCI or CABG	7744(8.4)	2984(7.0)	3110 (9.3)	804(9.6)	508(10.2)	338(10.6)	<0.001	
Family history of CAD	2481(2.7)	1354(3.2)	825 (2.5)	159(1.9)	78(1.6)	65(2.0)	<0.001	
Previous AF	2229(2.4)	409(1.0)	963 (2.9)	376(4.5)	283(5.7)	198(6.2)	<0.001	
Hypertension	61,096(65.3)	25,677 (60.4)	22,873 (68.4)	6194(73.9)	3774(75.8)	2578(81.2)	<0.001	
Hyperlipidemia	79,418(85.9)	37,122 (87.3)	28,639 (85.6)	7036(83.9)	4124(82.8)	2497(78.6)	<0.001	
Diabetes	27,582(29.8)	11,896(28.0)	9468 (28.3)	2842(33.9)	1949(39.1)	1427(44.9)	<0.001	
Previous stroke	8387(9.1)	2640(6.2)	3348 (10.0)	1128(13.5)	763(15.3)	508(16.0)	<0.001	
Previous heart failure	2070(2.2)	296 (0.7)	769 (2.3)	370(4.4)	312(6.3)	323(10.2)	<0.001	
Cigarette smoking	40,334(43.6)	22,059 (51.9)	13,229 (39.5)	2718(32.4)	1482(29.8)	846(26.6)	<0.001	
Statins before admission	16,304(17.6)	6817(16.0)	6184 (18.5)	1553(18.5)	1043(20.9)	707(22.3)	<0.001	
Antiplatelet drugs before admission	22,659(24.5)	9602(22.6)	8556 (25.6)	2187(26.1)	1369(27.5)	945(29.8)	<0.001	
β-blocker before admission	8873(9.6)	3599(8.5)	3449(10.3)	876(10.4)	534(10.7)	415(13.1)	<0.001	
ACEI/ARB before admission	9696(10.5)	3662(8.6)	3819(11.4)	1146(13.7)	674(13.5)	395(12.4)	<0.001	
Heart failure on admission	6610(7.1)	1545(3.6)	2449 (7.3)	1046(12.5)	845(17.0)	725(22.8)	<0.001	
Cardiac arrest on admission	1449(1.6)	455 (1.1)	529 (1.6)	197(2.3)	165(3.3)	103(3.2)	<0.001	
Cardiogenic shock on admission	2592(2.7)	625 (1.5)	856 (2.6)	426(5.1)	356(7.1)	239(7.5)	<0.001	
SBP on admission (mmHg)	130.7±23.5	130.1±22.0	131.2±23.5	130.4±25.5	130.0±27.5	133.2±29.4	0.001	
DBP on admission (mmHg)	78.3±14.4	79.8±14.0	77.6±14.0	76.3±15.2	75.3±15.7	75.9±16.6	<0.001	
h on admission (bpm)	77.5±16.2	76.6±14.6	77.1±16.2	79.2±18.8	81.3±20.3	82.6±20.7	<0.001	
Killip class≥3	9656 (10.4)	2301 (5.4)	2575 (10.7)	1551(18.5)	1210(24.3)	1019(32.1)	<0.001	
eGFR on admission (ml/min·1.73m^2^)	88.0 (68.3-99.8)	100.8(95.3-107.8)	78.8(70.3-85.1)	53.55 (49.50-56.90)	38.83 (34.88-42.13)	21.06 (13.97-26.00)	<0.001	
HBA1C, (%)	6.1(5.6-7.2)	6.0 (5.6-7.3)	6.0 (5.6-7.0)	6.2 (5.7-7.3)	6.3 (5.7-7.6)	6.4(5.8-7.6)	<0.001	
HB on admission (g/L)	137.84±19.40	136.0±20.5	135.0±19.3	129.7±20.6	124.7±22.0	113.5±25.6	<0.001	
TC (mmol/L)	4.43±1.23	4.51±1.24	4.39±1.21	4.37±1.25	4.32±1.29	4.24±1.36	<0.001	
TG (mmol/L)	1.44 (1.02-2.14)	1.52(1.07-2.26)	1.38(0.98-2.02)	1.38(0.97-2.03)	1.38(0.95-2.09)	1.42(1.02-2.11)	<0.001	
LDL-C (mmol/L)	2.69 (2.08-3.35)	2.71 (2.12-3.35)	2.63 (2.05-3.27)	2.60 (2.00-3.25)	2.55 (1.95-3.24)	2.43 (1.82-3.13)	<0.001	
HDL-C (mmol/L)	1.01 (0.85-1.22)	1.03 (0.86-1.24)	1.05 (0.87-1.26)	1.04 (0.86-1.27)	1.03 (0.85-1.27)	1.00 (0.80-1.23)	<0.001	
Undergoing CA	74,241 (80.3)	36,514 (85.9)	26,821 (80.2)	6050 (72.2)	3196(64.2)	1660 (52.3)	<0.001	
Undergoing PCI	63,852(69.0)	32,026 (75.3)	22,799 (68.1)	5070(60.5)	2611(52.4)	1346(42.4)	<0.001	
Coronary artery lesions								
One-vessel lesion	42,416(57.1)	22,254(60.9)	14,695 (54.8)	3143 (51.9)	1558 (48.7)	766 (46.1)	<0.001	
multi-vessel lesions	27,946(37.6)	12,789(35.0)	10,545 (39.3)	2497 (41.3)	1381 (43.2)	734 (44.2)	<0.001	
Left main vessel lesions	3879(5.2)	1471(4.1)	1581 (5.9)	410(6.8)	257(8.0)	160(9.6)	<0.001	

Values are mean±SD, median(interquartile range, IQ), or n (%)

*CKD* chronic kidney disease; *eGFR* estimated glomerular filtration rate; *ACS *acute coronary syndrome; *STEMI *ST-elevation myocardial infarction; *NSTEMI *Non-ST elevation myocardial infarction; *UA *Unstable angina pectoris; *PCI *percutaneous coronary intervention; *CABG *coronary artery bypass grafting, *SBP* systolic blood pressure; *DBP* diastolic blood pressure; *HR* heart rate; *ACEI *Angiotensin Converting Enzyme Inhibitors; *ARB *Angiotensin receptor antagonist; *FBG* fasting blood glucose; *HBA1C* glycated hemoglobin; *HB *Hemoglobin; *TG *triglyceride; *TC *total cholesterol; *LDL-C *low-density lipoprotein cholesterol; *HDL *high-density lipoprotein cholesterol

The percentages of patients with eGFR≥90 ml/min·1.73m^2^, 60-89 ml/min·1.73m^2^, 45-59 ml/min 1.73 m^2^, 30-44 ml/min 1.73 m^2^ and < 30 ml/min 1.73 m^2^ were 45.95%, 36.17%, 9.06%, 5.38% and 3.43%, respectively. The comparison results were shown in Table 1. With the decrease of eGFR, the proportion of STEMI decreased gradually, while the proportion of NSTE-ACS increased gradually. The proportion of patients with hypertension, diabetes mellitus, heart failure, previous heart myocardial and previous stroke increased gradually, while the proportion of patients with hyperlipidemia and LDL-C level decreased gradually. With the decrease of eGFR, the proportion of patients with taking β-blockers, antiplatelet drugs and statins before admission increased, while the proportion of patients with taking ACEI/ARB decreased when the eGFR<60 ml/min·1.73m^2^. With the decrease of eGFR, the proportion of patients undergoing coronary angiography and PCI decreased significantly after admission, while the severity of coronary artery lesions in patients undergoing angiography increased gradually.

**Fig. 1:**
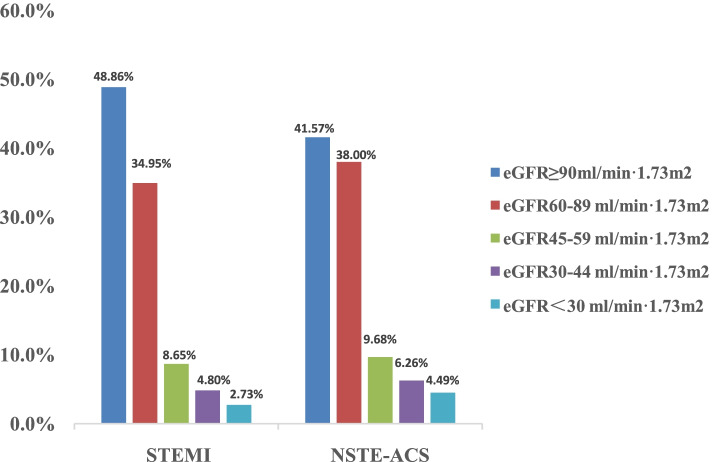
Percentage of different renal function in STEMI and NSTE-ACS patients. eGFR: estimated glomerular filtration rate;STEMI: ST-elevation myocardial infarction, NSTE-ACS, Non-ST elevation acute coronary syndrome

Compared to the patients with STEMI, patients with NSTE-ACS had a higher proportion of RI: the percentages of patients with 60-89 ml/min·1.73m^2^,45-59 ml/min 1.73 m^2^, 30-44 ml/min 1.73 m^2^ and < 30 ml/min 1.73 m^2^ in STEMI patients were 34.95%, 8.65%, 4.89% and 2.73%, respectively, while those in NSTE-ACS patients were 38.00%, 9.68%, 6.26% and 4.49%, respectively. (Figure 1) Compared to the patients with STEMI, the proportion of previous MI, hypertension, diabetes, previous stroke, previous PCI or CABG, use of β-blocker and ACEI/ARB before admission were increased in patients with NSTE-ACS.(Additional file: Table S3).

### In-hospital outcomes

**Fig. 2:**
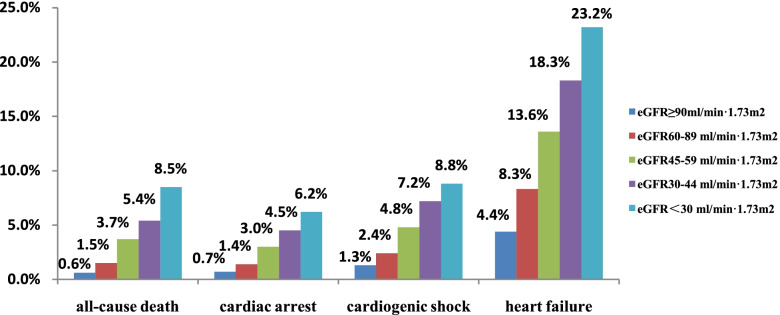
Incidence of MACEs in patients with ACS under different renal functions. eGFR: estimated glomerular filtration rate

The proportion of in-hospital mortality was 1.7%, heart failure in hospital was 8.0%, cardiogenic shock in hospital was 2.6%, and cardiac arrest in hospital was 1.6% in all the ACS patients. Moreover, the proportion of MACEs were 7.0%, 13.6%, 25.1%, 35.4%, and 46.7% in patients with eGFR ≥ 90ml/min·1.73m^2^, 60-89 ml/min·1.73m^2^, 45-59 ml/min 1.73 m^2^, 30-44 ml/min 1.73 m^2^ and < 30 ml/min 1.73 m^2^ respectively. With the decrease of eGFR, the proportion of in-hospital mortality, heart failure, cardiac arrest and cardiogenic shock increased gradually. (Figure 2).

Compared to the patients with STEMI, patients with NSTE-ACS had a higher proportion of all-cause death, cardiac arrest, cardiogenic shock, heart failure were decreased.(Additional file: Table S4).

### Relationship between eGFR and MACEs

To evaluate the association between eGFR and MACEs, logistic regression analyses were performed in the ACS population. In univariate logistic regression analysis, a significantly higher risk of the MACEs was observed in patients with RI. After adjusting for confounders in the multivariate logistic regression model, patients with eGFR 60-89 ml/min·1.73m^2^, 45-59 ml/min·1.73m^2^, 30-44 ml/min·1.73m^2^ and <30 ml/min·1.73m^2^ had a 1.3-fold(OR, 1.27; 95% CI, 1.19-1.35), 1.7-fold(OR, 1.65; 95% CI, 1.52-1.80), 2.0-fold(OR, 2.04; 95% CI, 1.85-2.26) and 2.2-fold(OR, 2.23, 95% CI, 1.98-2.50) increased risk of MACEs compared to patients with eGFR ≥90 ml/min·1.73m^2^. (Table 2)

**Table 2:** Attributable risk of MACEs in different renal function in all the ACS patients

	Unadjusted			Adjusted			
	OR	95%CI	P	OR^a^	95%CI	P	AR
eGFR ≥90 ml/min·1.73m^2^	ref			ref			
60-89 ml/min·1.73m^2^	1.82	1.73-1.92	<0.001	1.27	1.19-1.35	<0.001	7.78%
45-59 ml/min·1.73m^2^	3.18	2.97-3.41	<0.001	1.65	1.52-1.80	<0.001	4.69%
30-44 ml/min·1.73m^2^	4.62	4.28-4.99	<0.001	2.04	1.85-2.26	<0.001	4.46%
<30 ml/min·1.73m^2^	6.06	5.55-6.61	<0.001	2.23	1.98-2.50	<0.001	3.36%

*MACEs* major adverse cardiovascular events; *ACS* acute coronary syndrome; *eGFR* estimated glomerular filtration rate

^a^ORs were adjusted for age, history of hypertension, diabetes mellitus, heart failure, atrial fibrillation, stroke, previous PCI or CABG, ACS type, heart failure at admission, cardiogenic shock at admission, cardiac arrest at admission, Killip class at admission, systolic pressure at admission, taking antiplatelet drugs before admission, taking β-blocker before admission and taking ACEI/ARB before admission, HB at admission, LDL-C at admission

In order to explore the relationship between eGFR and MACEs in different types of ACS, subgroup analysis was performed based on STEMI and NST-ACS population respectively. In the patients with STEMI, after adjusted for confounders in the multivariate logistic regression model, patients with eGFR 60-89 ml/min·1.73m^2^, 45-59 ml/min·1.73m^2^, 30-44 ml/min·1.73m^2^ and <30 ml/min·1.73m^2^ had a 1.3-fold(OR, 1.25; 95% CI, 1.16-1.34), 1.6-fold(OR, 1.56; 95% CI, 1.40-1.73), 1.9-fold(OR, 1.92; 95% CI, 1.70-2.17) and 2.4-fold(OR, 2.38, 95% CI, 2.05-2.76) increased risk of MACEs compared to patients with eGFR ≥90 ml/min·1.73m^2^. (Table 3) In the patients with NST-ACS, after adjusting for confounders in the multivariate logistic regression model, patients with eGFR 60-89 ml/min·1.73m^2^, 45-59 ml/min·1.73m^2^, 30-44 ml/min·1.73m^2^ and <30 ml/min·1.73m^2^ had a 1.4-fold(OR, 1.41; 95% CI, 1.25-1.59), 2.0-fold(OR, 12.01; 95% CI, 1.72-2.34), 2.6-fold(OR, 2.57; 95% CI, 2.18-3.04) and 2.7-fold(OR, 2.69, 95% CI, 2.23-3.24) increased risk of MACEs compared to patients with eGFR ≥90 ml/min·1.73m^2^. (Table 3)

**Table 3:** Attributable risk of MACEs in different renal function in STEMI and NSTE-ACS patients

	STEMI patients				NSTE-ACS patients			
	Adjusted OR (95% CI)		P	AR	Adjusted OR^a^ (95% CI)		P	AR
eGFR ≥90 ml/min·1.73m^2^	ref				ref			
60-89 ml/min·1.73m^2^	1.25	1.16-1.34	<0.001	7.18%	1.41	1.25-1.59	<0.001	10.91%
45-59 ml/min·1.73m^2^	1.56	1.40-1.73	<0.001	3.98%	2.01	1.72-2.34	<0.001	6.85%
30-44 ml/min·1.73m^2^	1.92	1.70-2.17	<0.001	3.63%	2.57	2.18-3.04	<0.001	6.88%
<30 ml/min·1.73m^2^	2.38	2.05-2.76	<0.001	3.09%	2.69	2.23-3.24	<0.001	5.31%

*MACEs* major adverse cardiovascular events; *STEMI* ST segment elevated myocardial infarction; *NSTE-ACS *non-ST segment elevated acute coronary syndrome; *eGFR *estimated glomerular filtration rate

^a^ORs were adjusted for age, history of hypertension, diabetes mellitus, heart failure, atrial fibrillation, stroke, previous PCI or CABG, ACS type, heart failure at admission, cardiogenic shock at admission, cardiac arrest at admission, Killip class at admission, systolic pressure at admission, taking antiplatelet drugs before admission, taking β-blocker before admission and taking ACEI/ARB before admission, HB at admission, LDL-C at admission

### The attributable risk of eGFR for MACEs

In all the ACS patients, the attributable risk of eGFR 60-89 ml/min·1.73m^2^ was 7.78%, 4.69% of eGFR 45-59 ml/min·1.73m^2^, 4.46% of eGFR 30-44 ml/min·1.73m^2^, and 3.36% of eGFR<30 ml/min·1.73m^2^. (Table 2)

According to the results of regression analysis, we calculated the attributable risk of MACEs in STEMI and NST-ACS population with different eGFR stratification. In the patients with STEMI, the attributable risk of eGFR 60-89 ml/min·1.73m^2^ was 7.18%, 3.98% of eGFR 45-59 ml/min·1.73m^2^, 3.63% of eGFR 30-44 ml/min·1.73m^2^, and 3.09% of eGFR<30 ml/min·1.73m^2^. In the patients with NSTE-ACS, the attributable risk of eGFR 60-89 ml/min·1.73m^2^ for MACEs was 10.91%, 6.85% of eGFR 45-59 ml/min·1.73m^2^, 6.88% of eGFR 30-44 ml/min·1.73m^2^, and 5.31% of eGFR<30 ml/min·1.73m^2^.(Table 3).

## Discussion

In this large, hospital-based registry for patients with ACS, eGFR was significantly associated with the risk of MACEs during hospitalization. Moreover, the attributable risk of mild RI to MACEs was higher than that of moderate to severe RI, which is especially obvious in patients with NSTE-ACS.

The subjects of this study were patients with ACS. In the present study, we found the proportion of patients with eGFR < 90 ml / min · 1.73m^2^ was 54.05%, and the proportion of patients with mild RI (eGFR 60-89 ml / min · 1.73m^2^) was 36.17%. This was similar to the foreign literature report: in a study including 20,604 patients with ACS in New Zealand, 53.3%, 23.3%, 1.7% and 1.4% of patients combined with CKD stages 2, 3, 4 and 5 respectively [23]. In other studies with relative small sample size, the proportion of ACS patients with RI is also similar [24, 25]. It can be seen that the proportion of patients with RI is large in ACS population and which is mainly mild RI.

The present study also found that RI was an independent risk factor for MACEs during hospitalization, and there was a gradual correlation between them. In the multivariate regression analysis, the odds ratio(95% CI) of MACEs in the patients with 60-89 ml/min·1.73m^2^,45-59 ml/min·1.73m^2^, 30-44 ml/min·1.73m^2^ and <30 ml/min·1.73m^2^ were 1.27, 1.65, 2.04 and 2.23. We can see from this data that the worse the renal function in the patients with ACS, the higher risk of MACEs during hospitalization, which is consistent with the foreign literature reports [13, 26]. Therefore, we always pay more attention to the ASC patients with severe RI in clinical work, while often ignore the ACS patients with mild RI.

However, it also can be seen from the data of the present study that only the slightly decrease of eGFR (60-89 ml / min·1.73m^2^) in the patients with ACS, the risk of MACEs increased 1.27 times compared to those with normal renal function (eGFR ≥ 90ml / min·1.73m^2^). Smith GL et al. followed up 118,753 patients with AMI for 10 years. They found that even mild impairment of renal function (eGFR 66-74 ml/min.1.73 m^2^) could increase 10-year mortality risk of patients to 10% compared to patients with normal renal function [27]. El menyar et al. retrospectively analyzed 6518 patients with ACS and found that ACS patients with mild RI (eGFR 60-89 ml/min.1.73 m^2^) increased the risk of in-hospital mortality by 2.1 times compared with ACS patients with normal renal function[15].Other studies with relatively small sample size also found that mild RI was related to the short-term and long-term prognosis in ACS patients [27–29]. However, only regression analysis was used to analyze the relationship between mild RI and prognosis of ACS patients in these studies. And this was not be reported in previous literature that used attributable risk to reveal the relationship between mild RI and hospital outcomes in ACS population. Attributable risk is reflected in the total chance of a disease (or death) in the population exposed to a certain factor, the part that really attributable to the exposure factor. The public health significance of this index is that for the exposed population, if the exposure factors are eliminated, the number of morbidity (or death) per unit population can be reduced. In the present study, the attributable risk of eGFR 60-89ml/min·1.73m^2^ was 7.78%, eGFR 45-59 ml/min·1.73m^2^ was 4.69%, eGFR 30-44 ml/min·1.73m^2^ was 4.46% and eGFR<30 ml/min·1.73m^2^ was 3.36%. That is to say, among the ACS patients, 7.78% of MACEs during hospitalization was caused by mild RI. It can be seen that in the ACS population in the present study, although the risk of MACEs during hospitalization with mild RI was lower than that of moderate and severe RI, its attributable risk is far greater than that of moderate and severe RI. The occurrence of this phenomenon is attributed to the proportion of ACS patients with mild RI far greater than that of ACS patients with moderate and severe RI. For example, the patients with mild RI accounts for 36.17% of all the ACS patients in the present study. Therefore, even if the creatinine is slightly increased, we should pay enough attention and carry out early intervention to avoid the occurrence of adverse events in the hospital. Overall, attributable risks can allow for optimal allocation of resources toward the prevention of MACEs associated with ACS and suggest that primary prevention strategies may be needed at mild RI.

We even found that in the NSTE-ACS population, the attributable risk of eGFR 60-89 ml/min·1.73m^2^ to MACEs during hospitalization was as high as 10.91%. While that is 7.18% in patients with STEMI. In the present study, the proportion of NST-ACS patients with RI compared with STEMI patients with RI was 58.43% vs. 51.14%. Moreover, 34.95% of patients with STEMI had mild RI, while that of NST-ACS patients was 38%. It can be seen that patients with NSTE-ACS are more likely to have a poor basic renal function than patients with STEMI, which is similar to Gupta’s conclusion [13]. They analyzed 3,187,404 patients. In the ACS subgroup, the percentages of STEMI in non CKD, CKD and ESRD patients were 34.5%, 22.3% and 16.6% respectively, while the percentages of NSTE-ACS were 65.5%, 77.7% and 83.4%. The occurrence of this phenomenon may be related to the presence of microinflammation, vascular calcification caused by abnormal bone metabolism, etc. in patients with RI, which leads to the progression of chronic invasive plaque of coronary artery and chronic obstruction, eventually lead to NSTE-ACS[30, 31]. It is precisely because the basic renal function of patients with NSTE-ACS is worse than that of patients with STEMI, and the percentage of patients with mild RI is higher, so the attribution risk of mild RI to MACEs during hospitalization is higher in patients with NSET-ACS. This also suggests that once NSTE-ACS patients with mild RI, we need to be highly vigilant and actively prevent the occurrence of MACEs.

The current study has the following limitations: (1) Because cardiologists usually avoid to do coronary angiography in patients with RI because of a high risk. Therefore, the population for assessing the severity of coronary artery lesions in our study, may not be represented all the ACS patients. (2) Because the data in our study are obtained from CCC-ACS database, which does not be included the data of calcium and phosphorus metabolism, inflammation, the current study couldn’t adjust the impact of the above factors on the short-term outcomes in patients with ACS. (3) The outcome of our study was limited to hospital events and follow-up data were not available.

## Conclusions

In conclusion, the present study is the largest sample study for the ACS population in China. It is suggested that the mild decline of eGFR is an independent risk factor for the poor short-term prognosis in ACS patients. Moreover, the attributable risk of mild RI is far greater than that of moderate and severe RI in ACS patients with short prognosis during hospitalization, which is especially in NSTE-ACS patients. Therefore, in clinical work, the prevention of cardiovascular disease is not only limited to moderate and severe RI, but also start from those with early RI.

## Supplementary Information

**Additional file 1.**

## Data Availability

The datasets analyzed during the current study are not publicly available because of intellectual property rights, but are available from the Prof. Dong Zhao on reasonable request.

## References

[1] Ma LY, Chen WW, Gao RL, Liu LS, Zhu ML, Wang YJ (2020). China cardiovascular diseases report 2018: an updated summary. J Geriatr Cardiol.

[2] Moran A, Gu D, Zhao D, Coxson P, Wang YC, Chen CS (2010). Future cardiovascular disease in China: Markov model and risk factor scenario projections from the coronary heart disease policy model-China. Circ Cardiovasc Qual Outcomes.

[3] Smith SC, Zheng ZJ (2010). The impending cardiovascular pandemic in China. Circ Cardiovasc Qual Outcomes.

[4] Cheng J, Zhao D, Zeng Z, Critchley JA, Liu J, Wang W (2009). The impact of demographic and risk factor changes on coronary heart disease deaths in Beijing, 1999-2010. BMC Public Health.

[5] Moran A, Zhao D, Gu D, Coxson P, Chen CS, Cheng J (2008). The future impact of population growth and aging on coronary heart disease in China: projections from the coronary heart disease policy model-China. BMC Public Health.

[6] Wang S, Marquez P, Longenbrunner J (2011). Toward a healthy and harmonious life in China: stemming the rising tide of non-communicable diseases.

[7] Zhou M, Wang H, Zhu J, Chen W, Wang L, Liu S (2016). Cause-specific mortality for 240 causes in China during 1990–2013: a systematic subnational analysis for the Global Burden of Disease Study 2013. Lancet.

[8] Ibanez B, James S, Agewall S, Antunes MJ, Bucciarelli-Ducci C, Bueno H (2018). 2017 ESC guidelines for the management of acute myocardial infarction in patients presenting with ST-segment elevation: the task force for the management of acute myocardial infarction in patients presenting with ST-segment elevation of the European Society of Cardiology(ESC). Eur Heart J.

[9] Fihn SD, Gardin JM, Abrams J, Berra K, Blankenship JC, Dallas AP (2012). 2012 ACCF/AHA/ACP/AATS/PCNA/SCAI/STS guideline for the diagnosis and management of patients with stable ischemic heart disease: a report of the American College of Cardiology Foundation/American Heart Association Task Force on Practice Guidelines, and the American College of Physicians, American Association for Thoracic Surgery, Preventive Cardiovascular Nurses Association, Society for Cardiovascular Angiography and Interventions, and Society of Thoracic Surgeons. J Am Coll Cardiol.

[10] Campbell-Scherer DL, Green LA (2009). ACC/AHA guideline update for the management of ST-segment elevation myocardial infarction. Am Fam Physician.

[11] Kimura K, Kimura T, Ishihara M, Nakagawa Y, Nakao K, Miyauchi K (2019). JCS 2018 guideline on diagnosis and treatment of acute coronary syndrome. Circ J.

[12] Orvin K, Eisen A, Goldenberg I, Farkash A, Shlomo N, Gevrielov-Yusim N (2015). The proxy of renal function that most accurately predicts short- and long-term outcome after acute coronary syndrome. Am Heart J.

[13] Gupta T, Paul N, Kolte D, Harikrishnan P, Khera S, Aronow WS (2015). Association of chronic renal insufficiency with in-hospital outcomes after percutaneous coronary intervention. J Am Heart Assoc.

[14] Pun PH, Smarz TR, Honeycutt EF, Shaw LK, Al-Khatib SM, Middleton JP (2009). Chronic kidney disease is associated with increased risk of sudden cardiac death among patients with coronary artery disease. Kidney Int.

[15] El-Menyar A, Zubaid M, Sulaiman K, Singh R, Al Thani H, Akbar M, et al. In-hospital major clinical outcomes in patients with chronic renal insufficiency presenting with acute coronary syndrome: data from a Registry of 8176 patients. Mayo Clin Proc. 2010;85:332-340. 10.4065/mcp.2009.0513 PMC2848421 20360292

[16] AlFaleh HF, Alsuwaida AO, Ullah A, Hersi A, AlHabib KF, AlShahrani A (2012). Glomerular filtration rate estimated by the CKD-EPI formula is a powerful predictor of in-hospital adverse clinical outcomes after an acute coronary syndrome. Angiology.

[17] Kang YU, Jeong MH, Kim SW (2009). Impact of renal dysfunction on clinical outcomes of acute coronary syndrome. Yonsei Med J.

[18] Goldenberg I, Subirana I, Boyko V, Vila J, Elosua R, Permanyer-Miralda G (2010). Relation between renal function and outcomes in patients with non-ST-segment elevation acute coronary syndrome: real-world data from the European Public Health Outcome Research and Indicators Collection Project. Arch Intern Med.

[19] Hao Y, Liu J, Liu J, Smith SC, Huo Y, Fonarow GC (2016). Rationale and design of the Improving Care for Cardiovascular Disease in China(CCC) project: a national effort to prompt quality enhancement for acute coronary syndrome. Am Heart J.

[20] Ma YC, Zuo L, Chen JH, Luo Q, Yu XQ, Li Y (2006). Modified glomerular filtration rate estimating equation for Chinese patients with chronic kidney disease. J Am Soc Nephrol.

[21] Walter SD (1976). The estimation and interpretation of attributable risk in health research. Biometrics.

[22] Hanley JA (2001). A heuristic approach to the formulas for population attributable fraction. J Epidemiol Community Health.

[23] Szummer K, Lundman P, Jacobson SH, Schön S, Lindbäck J, Stenestrand U (2010). Relation between renal function, presentation, use of therapies and in-hospital complications in acute coronary syndrome: data from the SWEDEHEART register. J Intern Med.

[24] Wiviott 24Rhee JW, Scirica SD, Gibson BM, Murphy CM, Bonaca SA (2014). Clinical features, use of evidence-based therapies, and cardiovascular outcomes among patients with chronic kidney disease following non-ST-elevation acute coronary syndrome. Clin Cardiol.

[25] Kim IY, Hwang IH, Lee KN, Lee DW, Lee SB, Shin MJ (2013). Decreased renal function is an independent predictor of severity of coronary artery disease: an application of Gensini score. J Korean Med Sci.

[26] Osten MD, Ivanov J, Eichhofer J, Seidelin PH, Ross JR, Barolet A (2008). Impact of renal insufficiency on angiographic, procedural, and in-hospital outcomes following percutaneous coronary intervention. Am J Cardiol.

[27] Masoudi FA, Plomondon ME, Magid DJ, Sales A, Rumsfeld JS (2004). Renal insufficiency and mortality from acute coronary syndromes. Am Heart J.

[28] Dohi T, Miyauchi K, Okazaki S, Yokoyama T, Tamura H, Kojima T (2011). Long-term impact of mild chronic kidney disease in patients with acute coronary syndrome undergoing percutaneous coronary interventions. Nephrol Dial Transplant.

[29] Campbell NG, Varagunam M, Sawhney V, Ahuja KR, Salahuddin N, De Palma R (2012). Mild chronic kidney disease is an independent predictor of long-term mortality after emergency angiography angiography and primary percutaneous intervention in patients with ST-elevation myocardial infarction. Heart.

[30] Sarnak MJ, Amann K, Bangalore S, Cavalcante JL, Charytan DM, Craig JC (2019). Chronic Kidney Disease and Coronary Artery Disease: JACC State-of-the-Art Review. J Am Coll Cardiol.

[31] Shroff GR, Li S, Herzog CA (2017). Trends in Discharge Claims for Acute Myocardial Infarction among Patients on Dialysis. J Am Soc Nephrol.

