# Supplementary material for: Mild renal insufficiency and attributable risk of adverse In-hospital outcomes in patients with Acute Coronary Syndrome from the improving care for Cardiovascular Disease in China (CCC) project
Source: BMC Nephrol. 2022 Jan 13;23:29. doi: 10.1186/s12882-022-02663-4 (PMC8759171; doi:10.1186/s12882-022-02663-4)
Supplement: Supplementary file 1 — Additional file 1. [file 12882_2022_2663_MOESM1_ESM.docx]

Table S1. Investigators of CCC-ACS project.

| **ID** | **Hospitals** | **Territories** | **Provinces** | **City** | **Investigator** |
| --- | --- | --- | --- | --- | --- |
| 1 | Shanxi Cardiovascular Hospital | Northern China | Shanxi | Taiyuan | Bao Li |
| 2 | Nanjing Drum Tower Hospital, The Affiliated Hospital of Nanjing University Medical School | Eastern China | Jiangsu | Nanjing | Biao Xu, Guangshu Han |
| 3 | Hainan General Hospital | Southern China | Hainan | Haikou | Bin Li |
| 4 | The Second Hospital of Jilin University | Northeast China | Jilin | Changchun | Bin Liu |
| 5 | The 2nd Affiliated Hospital of Harbin Medical University | Northeast China | Heilongjiang | Harbin | Bo Yu |
| 6 | The Ninth Hospital Affiliated to Shanghai Jiaotong University School of Medicine | Eastern China | Shanghai | Shanghai | Changqian Wang |
| 7 | Henan Provincial People’s Hospital | Central China | Henan | Zhengzhou | Chuanyu Gao |
| 8 | Shanxi Provincial People’s Hospital | Northern China | Shanxi | Taiyuan | Chunlin Lai |
| 9 | Xinqiao Hospital, Third Military Medical University | Southwest China | Chongqing | Chongqing | Cui Bin, Lan Huang |
| 10 | China Meitan General Hospital | Northern China | Beijing | Beijing | Di Wu |
| 11 | The 309th Hospital of Chinese People’s Liberation Army | Northern China | Beijing | Beijing | Fakuan Tang, Jun Xiao |
| 12 | Zhongda Hospital, Southeast University | Eastern China | Jiangsu | Nanjing | Genshan Ma |
| 13 | The First Affiliated Hospital of Liaoning Medical University | Northeast China | Liaoning | Jinzhou | Guizhou Tao |
| 14 | Xinjiang Uygur Autonomous Region People’s Hospital | Northwest China | Xinjiang | Urumchi | Guoqing Li |
| 15 | Sir Run Run Shaw Hospital, College of Medicine, Zhejiang University | Eastern China | Zhejiang | Hangzhou | Guosheng Fu |
| 16 | Beijing Friendship Hospital, Capital Medical University | Northern China | Beijing | Beijing | Hongwei Li |
| 17 | The First Affiliated Hospital of Bengbu Medical College | Eastern China | Anhui | Bengbu | Honhju Wang |
| 18 | General Hospital of TISCO | Northern China | Shanxi | Taiyuan | Huifeng Wang |
| 19 | Dongguan People’s Hospital | Southern China | Guangdong | Dongguan | Jianfeng Ye |
| 20 | Panyu Hospital of Chinese Medicine | Southern China | Guangdong | Guangzhou | Jianhao Li |
| 21 | Peking University First Hospital | Northern China | Beijing | Beijing | Jie Jiang |
| 22 | Sun Yat-sen Memorial Hospital, Sun Yat-sen University | Southern China | Guangdong | Guangzhou | Jingfeng Wang |
| 23 | Guangdong General Hospital | Southern China | Guangdong | Guangzhou | Jiyan Chen |
| 24 | Hospital of Xinjiang Production & Construction Corps | Northwest China | Xinjiang | Urumchi | Junming Liu |
| 25 | The Military General Hospital of Beijing PLA | Northern China | Beijing | Beijing | Junxia Li |
| 26 | The First Affiliated Hospital of Guangxi Medical University | Southern China | Guangxi | Nanning | Lang Li |
| 27 | Tongren Hospital Affiliated to Shanghai Jiaotong University School of Medicine | Eastern China | Shanghai | Shanghai | Li Jiang |
| 28 | Binzou City Center Hospital | Eastern China | Shandong | Binzhou | Lijun Meng |
| 29 | The First Affiliated Hospital of Zhengzhou University | Central China | Henan | Zhengzhou | Ling Li |
| 30 | Xijing Hospital | Northwest China | Shaanxi | Xi’an | Ling Tao |
| 31 | The Affiliated Hospital of Guizhou Medical University | Southwest China | Guizhou | Guiyang | Lirong Wu |
| 32 | First Affiliated Hospital of the People’s Liberation Army General Hospital | Northern China | Beijing | Beijing | Miao Tian |
| 33 | The Second People’s Hospital of Yunnan Province | Southwest China | Yunnan | Kunming | Minghua Han |
| 34 | Haikou People’s Hospital | Southern China | Hainan | Haikou | Moshui Chen |
| 35 | Gansu Provincial Hospital | Northwest China | Gansu | Lanzhou | Ping Xie |
| 36 | The First Affiliated Hospital of Henan University of Science and Technology | Central China | Henan | Luoyang | Pingshuan Dong |
| 37 | Chenzhou First People’s Hospital | Central China | Hunan | Chenzhou | Qiaoqing Zhong |
| 38 | People’s Hospital of Qinghai Province | Northwest China | Qinghai | Xining | Rong Chang |
| 39 | Affiliated Hospital of Ningxia Medical University | Northwest China | Ningxia | Yinchuan | Shaobin Jia |
| 40 | Beijing Anzhen Hospital, Capital Medical University | Northern China | Beijing | Beijing | Shaoping Nie, Xiaohui Liu |
| 41 | North Jiangsu People’s Hospital | Eastern China | Jiangsu | Yangzhou | Shenghu He |
| 42 | Shanghai Sixth People’s Hospital | Eastern China | Shanghai | Shanghai | Shixin Ma |
| 43 | The First Hospital of Handan | Northern China | Hebei | Handan | Shuanli Xin |
| 44 | Huai’an First People’s Hospital | Eastern China | Jiangsu | Huai’an | Shuren Ma |
| 45 | The First Affiliated Hospital of Chongqing Medical University | Southwest China | Chongqing | Chongqing | Suxin Luo |
| 46 | Navy General Hospital | Northern China | Beijing | Beijing | Tianchang Li |
| 47 | Zhejiang Provincial Hospital of TCM | Eastern China | Zhejiang | Hangzhou | Wei Mao |
| 48 | The Third Xiangya Hospital of Central South University | Central China | Hunan | Changsha | Weihong Jiang |
| 49 | Affiliated Hospital of Qinghai University | Northwest China | Qinghai | Xining | Weijun Liu |
| 50 | Teda International Cardiovascular Hospital | Northern China | Tianjin | Tianjin | Wenhua Lin |
| 51 | The Second Hospital of Hebei Medical University | Northern China | Hebei | Shijiazhuang | Xianghua Fu |
| 52 | Changhai Hospital of Shanghai | Eastern China | Shanghai | Shanghai | Xianxian Zhao |
| 53 | The Second Affiliated Hospital to Nanchang University | Eastern China | Jiangxi | Nanchang | Xiaoshu Cheng |
| 54 | Hebei General Hospital | Northern China | Hebei | Shijiazhuang | Xiaoyong Qi |
| 55 | Inner Mongolia People’s Hospital | Northern China | Inner Mongolia | Hohhot | Xingsheng Zhao |
| 56 | The General Hospital of Shenyang Military Region | Northeast China | Liaoning | Shenyang | Yaling Han |
| 57 | The First Hospital of Jilin University | Northeast China | Jilin | Changchun | Yang Zheng |
| 58 | Tianjin Chest Hospital | Northern China | Tianjin | Tianjin | Yin Liu |
| 59 | Hunan Provincial People’s Hospital | Central China | Hunan | Changsha | Ying Guo |
| 60 | People’s Hospital of Yuxi City | Southwest China | Yunnan | Yuxi | Yinglu Hao |
| 61 | The People’s Hospital of Guangxi Zhuang Autonomous Region | Southern China | Guangxi | Nanning | Yingzhong Lin |
| 62 | The First Teaching Hospital of Xinjiang Medical University | Northwest China | Xinjiang | Urumchi | Yitong Ma |
| 63 | Baogang Hospital | Northern China | Inner Mongolia | Baotou | Yongdong Li |
| 64 | Tianjin Medical University General Hospital | Northern China | Tianjin | Tianjin | Yuemin Sun |
| 65 | The Second Affiliated Hospital of Zhengzhou University | Central China | Henan | Zhengzhou | Yulan Zhao |
| 66 | Nanfang Hospital of Southern Medical University | Southern China | Guangdong | Guangzhou | Yuqing Hou |
| 67 | The First Affiliated Hospital to Nanchang University | Eastern China | Jiangxi | Nanchang | Zeqi Zheng |
| 68 | The First Affiliated Hospital of Lanzhou University | Northwest China | Gansu | Lanzhou | Zheng Zhang |
| 69 | The Third Hospital of Shijiazhuang | Northern China | Hebei | Shijiazhuang | Zhenguo Ji |
| 70 | Wuxi People’s Hospital | Eastern China | Jiangsu | Wuxi | Zhenyu Yang |
| 71 | Jiangsu Province Hospital | Eastern China | Jiangsu | Nanjing | Zhijian Yang |
| 72 | The Second Hospital of Shanxi Medical University | Northern China | Shanxi | Taiyuan | Zhiming Yang |
| 73 | The Affiliated Hospital of Xuzhou Medical College | Eastern China | Jiangsu | Xuzhou | Zhirong Wang |
| 74 | Southwest Hospital, Third Military Medical University | Southwest China | Chongqing | Chongqing | Zhiyuan Song |
| 75 | The First Affiliated Hospital of Xi’an Jiaotong University | Northwest China | Shaanxi | Xi’an | Zuyi Yuan |
| 76 | Yangzhou First People’s Hospital | Eastern China | Jiangsu | Yangzhou | Aihua Li |
| 77 | Hospital 463 of Chinese People’s Liberation Army | Northeast China | Liaoning | Shenyang | Bosong Yang |
| 78 | The Central Hospital of Mianyang | Northwest China | Sichuan | Mianyang | Caidong Luo |
| 79 | Liaocheng People’s Hospital | Eastern China | Shandong | Liaocheng | Chunyan Zhang |
| 80 | Yancheng Third People’s Hospital | Eastern China | Jiangsu | Yancheng | Chunyang Wu |
| 81 | The Second Xiangya Hospital of Central South University | Central China | Hunan | Changsha | Daoquan Peng |
| 82 | The Central Hospital of Panzhihua | Northwest China | Sichuan | Panzhihua | Dawen Xu |
| 83 | The First Hospital of Qiqihar City | Northeast China | Heilongjiang | Qiqihar | Gang Xu |
| 84 | The Third the People’s Hospital of Bengbu | Eastern China | Anhui | Bengbu | Gengsheng Sang |
| 85 | The First Hospital of Jiamusi | Northeast China | Heilongjiang | Jiamusi | Guixia Zhang |
| 86 | Zhoushan People’s Hospital | Eastern China | Zhejiang | Zhoushan | Guoxiong Chen |
| 87 | Dalian Municipal Central Hospital | Northeast China | Liaoning | Dalian | Hailong Lin |
| 88 | Renmin Hospital of Wuhan University | Central China | Hubei | Wuhan | Hong Jiang |
| 89 | Ningxia People’s Hospital | Northwest China | Ningxia | Yinchuan | Hong Luan |
| 90 | The First People’s Hospital of Yunnan Province (Kunhua Hospital) | Southwest China | Yunnan | Kunming | Hong Zhang |
| 91 | The Central Hospital of Zhoukou | Central China | Henan | Zhoukou | Hualing Liu |
| 92 | Anyang District Hospital | Central China | Henan | Anyang | Hui Liu |
| 93 | Sichuan Provincial People’s Hospital | Northwest China | Sichuan | Chengdu | Jianhong Tao |
| 94 | Mudanjiang Cardiovascular Disease Hospital | Northeast China | Heilongjiang | Mudanjiang | Jianwen Liu |
| 95 | Yichang Central Hospital | Central China | Hubei | Yichang | Jiawang Ding |
| 96 | Qilu Hospital of Shandong University | Eastern China | Shandong | Jinan | Jifu Li |
| 97 | Affiliated Hospital of Jiangsu University | Eastern China | Jiangsu | Zhenjiang | Jinchuan Yan |
| 98 | The First People’s Hospital of Nanning City | Southern China | Guangxi | Nanning | Jinru Wei |
| 99 | The First Affiliated Hospital of Fujian Medical University | Eastern China | Fujian | Fuzhou | Jinzi Su |
| 100 | Chengdu Third People’s Hospital | Northwest China | Sichuan | Chengdu | Jiong Tang |
| 101 | Yantaishan hospital | Eastern China | Shandong | Yantai | Juexin Fan |
| 102 | Qingdao Municipal Hospital | Eastern China | Shandong | Qingdao | Jun Guan |
| 103 | Zhongshan Hospital Affiliated to Fudan University | Eastern China | Shanghai | Shanghai | Junbo Ge |
| 104 | Longyan First Hospital | Eastern China | Fujian | Longyan | Kaihong Chen |
| 105 | Affiliated Hospital of Guangdong Medical College | Southern China | Guangdong | Guangzhou | Keng Wu |
| 106 | Jiangxi Provincial People’s Hospital | Eastern China | Jiangxi | Nanchang | Lang Ji |
| 107 | Anhui Provincial Hospital | Eastern China | Anhui | Hefei | Likun Ma |
| 108 | Xiangtan City Central Hospital | Central China | Hunan | Xiangtan | Lilong Tang |
| 109 | The First Hospital of Haerbin City | Northeast China | Heilongjiang | Harbin | Lin Wei |
| 110 | Central Hospital Affiliated to Shenyang Medical College | Northeast China | Liaoning | Shenyang | Man Zhang, Kaiming Chen |
| 111 | The Central Hospital of Wuhan | Central China | Hubei | Wuhan | Manhua Chen |
| 112 | Hangzhou First People’s Hospital | Eastern China | Zhejiang | Hangzhou | Ningfu Wang |
| 113 | The Central Hospital of Xuzhou | Eastern China | Jiangsu | Xuzhou | Peiying Zhang |
| 114 | The Second hospital of Dalian Medical University | Northeast China | Liaoning | Dalian | Peng Qu |
| 115 | The First Affiliated Hospital of Liaoning University of Traditional Chinese Medicine | Northeast China | Liaoning | Shenyang | Ping Hou |
| 116 | Beijing Tsinghua Changgung Hospital | Northern China | Beijing | Beijing | Ping Zhang |
| 117 | Guizhou Provincial People’s Hospital | Southwest China | Guizhou | Guiyang | Qiang Wu |
| 118 | The First Affiliated Hospital of Xiamen University | Eastern China | Fujian | Xiamen | Qiang Xie |
| 119 | Quanzhou First Hospital | Eastern China | Fujian | Quanzhou | Rong Lin |
| 120 | Wuzhou People’s Hospital | Southern China | Guangxi | Wuzhou | Shaowu Ye |
| 121 | The Central Hospital of Jilin | Northeast China | Jilin | Changchun | Shuangbin Li |
| 122 | Xiangya Hospital Central South University | Central China | Hunan | Changsha | Tianlun Yang |
| 123 | Guangzhou Red Cross Hospital | Southern China | Guangdong | Guangzhou | Tongguo Wu |
| 124 | The First Affiliated Hospital of Guangzhou Medical College | Southern China | Guangdong | Guangzhou | Wei Wang |
| 125 | The First Affiliated Hospital of Wenzhou Medical University | Eastern China | Zhejiang | Wenzhou | Weijian Huang |
| 126 | The Second Affiliated Hospital of Soochow University | Eastern China | Jiangsu | Suzhou | Weiting Xu |
| 127 | Wuhan Asia Heart Hospital | Central China | Hubei | Wuhan | Xi Su |
| 128 | The First Affiliated Hospital of Soochow University | Eastern China | Jiangsu | Suzhou | Xiangjun Yang |
| 129 | Affiliated Hospital of Yan’an University | Northwest China | Shaanxi | Yan’an | Xiaochuan Ma |
| 130 | The First People’s Hospital of Jining | Eastern China | Shandong | Jining | Xiaofei Sun |
| 131 | The Central Hospital of Taiyuan | Northern China | Shanxi | Taiyuan | Xiaoping Chen |
| 132 | West China Hospital of Sichuan University | Northwest China | Sichuan | Chengdu | Xiaoping Chen |
| 133 | The Third Affiliated Hospital of Guangzhou Medical College | Southern China | Guangdong | Guangzhou | Ximing Chen |
| 134 | The First Affiliated Hospital of Wannan Medical College | Eastern China | Anhui | Wuhu | Xingsheng Tang |
| 135 | Tangdu Hospital of The Fourth Military Medical University | Northwest China | Shaanxi | Xi’an | Xue Li |
| 136 | Shanghai East Hospital Affiliated to Tongji University | Eastern China | Shanghai | Shanghai | Xuebo Liu |
| 137 | Xiamen Cardiovascular Disease Hospital | Eastern China | Fujian | Xiamen | Yan Wang |
| 138 | Zhongnan hospital of Wuhan University | Central China | Hubei | Wuhan | Yanggan Wang |
| 139 | Fujian Provincial Hospital | Eastern China | Fujian | Fuzhou | Yansong Guo |
| 140 | The First Affiliated hospital of Dalian Medical University | Northeast China | Liaoning | Dalian | Yanzong Yang |
| 141 | The First People’s Hospital of Changde | Central China | Hunan | Changde | Yi Huang |
| 142 | The First Affiliated Hospital of China Medical University | Northeast China | Liaoning | Shenyang | Yingxian Sun |
| 143 | The Fourth Affiliated Hospital of China Medical University | Northeast China | Liaoning | Shenyang | Yuanzhe Jin |
| 144 | Cangzhou Central Hospital | Northern China | Hebei | Cangzhou | Zesheng Xu |
| 145 | The Central Hospital of Shaoyang | Central China | Hunan | Shaoyang | Zewei Ouyang |
| 146 | The People’s Hospital of Liaoning Province | Northeast China | Liaoning | Shenyang | Zhanquan Li |
| 147 | The First Affiliated Hospital of Jiamusi University | Northeast China | Heilongjiang | Jiamusi | Zhaofa He |
| 148 | Tangshan Gongren Hospital | Northern China | Hebei | Tangshan | Zheng Ji |
| 149 | Huaibei Miners General Hospital | Eastern China | Anhui | Huaibei | Zhenqi Su |
| 150 | Linyi People’s Hospital | Eastern China | Shandong | Linyi | Zhihong Ou |
| 151 | Chongqing Hechuan District People’s Hospital | Southwest China | Chongqing | Chongqing | Xin Tang |
| 152 | Yuzhou City Central Hospital | Central China | Henan | Xuchang | Qinfeng Su |
| 153 | Jianshui County People’s Hospital | Southwest China | Yunnan | Honghe | Weiqing Fan |
| 154 | Dunhua City Hospital | Northeast China | Jilin | Dunhua | Fanju Meng |
| 155 | Shenyang City Electricity Central Hospital | Northeast China | Liaoning | Shenyang | Jing Xu |
| 156 | Shanghai Jingan District Shibei Hospital | Eastern China | Shanghai | Shanghai | Bin Wang |
| 157 | Beijing Fangshan District First Hospital | Northern China | Beijing | Beijing | Xuemei Peng |
| 158 | Hebei Daming County People’s Hospital | Northern China | Hebei | Handan | Haiping Guo |
| 159 | Jiangsu Binhai County People’s Hospital | Eastern China | Jiangsu | Yancheng | Yonglin Zhang |
| 160 | The First People’s Hospital of Longquanyi District | Southwest China | Sichuan | Chengdu | Wei Tuo |
| 161 | Guangxi Hengxian County People’s Hospital | Southern China | Guangxi | Nanning | Xianan Zhang |
| 162 | Hunan Changsha County First People’s Hospital | Central China | Hunan | Changsha | Siding Wang |
| 163 | People’s Hospital of Wugang | Central China | Hunan | Shaoyang | JiaoMei Yang |
| 164 | Longhui County People’s Hospital | Central China | Hunan | Shaoyang | Xiaojun Wang |
| 165 | Heilongjiang Fujin City Central Hospital | Northeast China | Heilongjiang | Jiamusi | Jiyan Yin |
| 166 | Dalian Fourth People’s Hospital | Northeast China | Liaoning | Dalian | Huifang Zhang |
| 167 | General Hospital of Guangzhou Military Command | Southern China | Guangdong | Guangzhou | Yanlie Zheng |
| 168 | The First People’s Hospital of Horqin District, Tongliao City | Northern China | Inner Mongolia | Tongliao | Junping Fang |
| 169 | Guiyang Sixth People’s Hospital | Southwest China | Guizhou | Guiyang | Kalan Luo |
| 170 | Geological Mining Hospital of Hunan Province | Central China | Hunan | Changsha | Naiyi Liang |
| 171 | Zhangzhou Municipal Hospital of Fujian Province | Eastern China | Fujian | Zhangzhou | Changyong Liu |
| 172 | Jining City Yanzhou District People’s Hospital | Eastern China | Shandong | Jining | Jian Yang |
| 173 | The People’s Hospital Feixian | Eastern China | Shandong | Linyi | Honghua Deng |
| 174 | Tangshan City Fengrun District People’s Hospital | Northern China | Hebei | Tangshan | Lin Wang |
| 175 | Qian’an People’s Hospital | Northern China | Hebei | Tangshan | Yuheng Yang |
| 176 | Yuzhong County People’s Hospital | Northwest China | Gansu | Lanzhou | Xiaowei Peng |
| 177 | Baiyin Cite Center Hospital | Northwest China | Gansu | Baiyin | Fang Zhao |
| 178 | Mingguang People’s Hospital | Eastern China | Anhui | Chuzhou | Yong Li |
| 179 | Xihua County People’s Hospital | Central China | Henan | Zhoukou | Chuntong Wang |
| 180 | Zhalantun People’s Hospital | Northern China | Inner Mongolia | Hulunbeier | Yuhua Zhu |
| 181 | Fengrun District Second People’s Hospital | Northern China | Hebei | Tangshan | Jingshan Zhao |
| 182 | Zhangping City Hospital | Eastern China | Fujian | Zhangpin | Jinxing Yi |
| 183 | Fuqing Cite Hospital | Eastern China | Fujian | Fuqing | Ping Chen |
| 184 | The Eight Affiliated Hospital, Sun Yat-sen University | Southern China | Guangdong | Guangzhou | Nan Jia |
| 185 | The Second Affiliated Hospital of Qiqihar Medical University | Northeast China | Heilongjiang | Qiqihar | Yanli Wang |
| 186 | Wuhan University of Science and Technology Hospital | Central China | Hubei | Wuhan | Jing Hu |
| 187 | Baotou City Center Hospital | Northern China | Inner Mongolia | Baotou | Ruiping Zhao |
| 188 | Shanghai Jiading District Center Hospital | Eastern China | Shanghai | Shanghai | Xia Chen |
| 189 | Datong City Second People’s Hospital | Northern China | Shanxi | Datong | Xiaoqin Zhang |
| 190 | Binyang People’s Hospital | Southern China | Guangxi | Binyang | Fudong Gan |
| 191 | Deqing People’s Hospital | Eastern China | Zhejiang | Deqing | Fangfang Huang |
| 192 | Xinmi people’s hospital | Central China | Henan | Xinmi | Xiaolei Li |
| 193 | Dongguan Changping hospital | Southern China | Guangdong | Dongguan | Haiyun Lin |
| 194 | Gongyi people’s hospital | Central China | Henan | Gongyi | Tianmin Du |
| 195 | Ye County people’s hospital | Central China | Henan | Yexian | Jie Yang |
| 196 | The second people’s hospital of Mengcheng | Eastern China | Anhui | Mengcheng | Pengfei Zhang |
| 197 | Nanpi People’s Hospital | Northern China | Hebei | Nanpi | Hui Dong |
| 198 | Shimen People’s Hospital | Central China | Hunan | Shimeng | Chuanliang Liang |
| 199 | Tieli People’s Hospital | Northeast China | Heilongjiang | Tieli | Yanbo Niu |
| 200 | Sihui People’s Hospital | Southern China | Guangdong | Sihui | Yuehua Huang |
| 201 | Chest Hospital of Xinjiang Uygur Autonomous Region | Northwest China | Xinjiang | Urumchi | Dongsheng Chai |
| 202 | Beian First People’s Hospital | Northeast China | Heilongjiang | Bei’an | Dongyan Li |
| 203 | Zunhua People’s Hospital | Northern China | Hebei | Zunhua | Xiaoli Yang |
| 204 | Lujiang People’s Hospital | Eastern China | Anhui | Lujiang | Qichun Wang |
| 205 | Qinyang People’s Hospital | Central China | Henan | Qinyang | Xiaowen Ma |
| 206 | Longmen People’s Hospital | Southern China | Guangdong | Longmen | Yingchao Luo |
| 207 | Quyang Renji Hospital | Northern China | Hebei | Quyang | Congliang Zhang |
| 208 | Nenjiang People’s Hospital | Northeast China | Heilongjiang | Nenjiang | Shuhua Zhang |
| 209 | Longjiang First People’s Hospital | Northeast China | Heilongjiang | Longjiang | Yuhuan Shi |
| 210 | Li County Hospital of Traditional Chinese Medicine | Central China | Hunan | Changde | Songbai Li |
| 211 | Luan County People’s Hospital | Northern China | Hebei | Luanxian | Guo Li |
| 212 | Yulong Hospital | Southwest China | Yunnan | Yulong | Zeyuan He |
| 213 | Huining People’s Hospital | Northwest China | Gansu | Huining | Jiabin Xi |
| 214 | Yuncheng Hospital | Eastern China | Shandong | Yuncheng | Jinglan Diao |
| 215 | Hepu People’s Hospital | Southern China | Guangxi | Hepu | Meisheng Lai |
| 216 | Duzishan Petrochemical Hospital | Northwest China | Xinjiang | Dushanzi | Shuqiu Qu |
| 217 | Guiding People’s Hospital | Southwest China | Guizhou | Guiding | Guoduo Chen |
| 218 | People’s Hospital of Rongchang District | Southwest China | Chongqing | Chongqing | Jie Chen |
| 219 | Ningbo First Hospital | Eastern China | Zhejiang | Ningbo | Huimin Chu |
| 220 | Ledong Second People’s Hospital | Southern China | Hainan | Ledong | Xiufeng Chen |
| 221 | Guang’an People’s Hospital | Southwest China | Sichuan | Guang’an | Tian Tuo |
| 222 | Linfen People’s Hospital | Northern China | Shanxi | Linfen | Junping Deng |
| 223 | People’s Hospital of Bozhou District | Southwest China | Guizhou | Zunyi | Shengyong Chen |
| 224 | Dianjiang People’s Hospital | Southwest China | Chongqing | Dianjiang | Yang Yu |
| 225 | First Affiliated Hospital of Harbin Medical University. | Northeast China | Heilongjiang | Harbin | Yue Li |
| 226 | Yiliang Hospital | Southwest China | Yunnan | Yiliang | Liqiong Yang |
| 227 | Haidong Ping’an District Hospital of Traditional Chinese Medicine | Northwest China | Qinghai | Haidong | Guoqin Xin |
| 228 | Ningjin People’s Hospital | Eastern China | Shandong | Ningjin | Tao Zhang |
| 229 | Yutian Hospital | Northern China | Hebei | Yutian | Xiaoyun Feng |
| 230 | Yanting People’s Hospital | Southwest China | Sichuan | Yanting | Mingcheng Bai |
| 231 | The Fourth Affiliated Hospital Zhejiang University School of Medicine | Eastern China | Zhejiang | Yiwu | Shudong Xia |
| 232 | Wuxi Xishan People’s Hospital | Eastern China | Jiangsu | Wuxi | Xudong Li |
| 233 | Dongfeng Hospital | Northeast China | Jilin | Dongfeng | Wei Liu |
| 234 | Zhijin People’s Hospital | Southwest China | Guizhou | Zhijin | Zhongshan Wang |
| 235 | Huaiyang People’s Hospital | Central China | Henan | Huaiyang | Li Wei |
| 236 | Suizhou Central Hospital | Central China | Hubei | Suizhou | Fengwei Li |
| 237 | Tonglu First People’s Hospital | Eastern China | Zhejiang | Tonglu | Xiaolan Li |
| 238 | Xiantao First People’s Hospital | Central China | Hubei | Xiantao | Dongmei Zhu |
| 239 | Honghu People’s Hospital | Central China | Hubei | Honghu | Hong Liu |
| 240 | Xinjin County Hospital of Traditional Chinese Medicine | Northwest China | Sichuan | Xinjin | Yingbi Su |

CCC-ACS: The Improving Care for Cardiovascular Disease in China-Acute Coronary Syndrome

Table S2. Variables with missing value and missing rates for total population in CCC - ACS project (N=92509).

| Variables | Missing rates, N (%) |
| --- | --- |
| Age | 361(0.39) |
| Systolic blood pressure | 161(0.17) |
| Diastolic blood pressure | 196(0.21) |
| Heart rate | 221(0.24) |
| Serum creatinine | 3671(3.97) |
| Hemoglobin | 3299(3.57) |
| Fasting plasma glucose | 12157(13.14) |
| Total cholesterol | 9695(10.48) |
| HDL-cholesterol | 9515(10.29) |
| LDL-cholesterol | 9109(9.85) |
| Triglyceride | 9295(10.05) |

CCC - ACS, The Improving Care for Cardiovascular Disease in China-Acute Coronary Syndrome; HDL, high density lipoprotein; LDL, low density lipoprotein

**Table S3** Baseline characteristics of STEMI and NSTE-ACS patients

| STEMI | | | | | | | | NSTE-ACS | | | | | | |
| --- | --- | --- | --- | --- | --- | --- | --- | --- | --- | --- | --- | --- | --- | --- |
|  | Total  （n=55574 ） | eGFR≥90ml/min·1.73m^2^  （n=27154,  48.86%） | eGFR60-89 ml/min·1.73m^2^  （n=19425,  34.95%） | eGFR45-59 ml/min·1.73m^2^  （n=4809,  8.65%） | eGFR30-44 ml/min·1.73m^2^  （n=2667,  4.80%） | eGFR＜  30 ml/min·1.73m^2^  （n=1519,  2.73%） | P value | Total  （n=36933 ） | eGFR≥90ml/min·1.73m^2^  （n=15352,  41.57%） | eGFR60-89 ml/min·1.73m^2^  （n=14037,  38.00%） | eGFR45-59 ml/min·1.73m^2^  （n=3574,  9.68%） | eGFR30-44 ml/min·1.73m^2^  （n=2313,  6.26%） | eGFR＜  30 ml/min·1.73m^2^  （n=1657,  4.49%） | P value |
| Male gender | 43251 (77.8) | 22743（83.8） | 14579（75.1） | 3279（68.2） | 1701（63.8） | 949（62.5） | ＜0.001 | 25119（68.0） | 11293（73.6） | 9416（67.1） | 1391（38.9） | 1009（43.6） | 734（44.3） | ＜0.001 |
| Age(years) | 61.89±12.64 | 55.70±10.59 | 66.49±11.36 | 70.21±11.71 | 71.42±11.71 | 70.60±12.00 | ＜0.001 | 65.19±11.89 | 58.12±9.74 | 68.81±10.36 | 72.54±10.23 | 73.67±10.90 | 72.31±11.45 | ＜0.001 |
| Previous MI | 3003（5.4） | 1238（4.6） | 1144 (5.9) | 318（6.6） | 188（7.0） | 115（7.4） | ＜0.001 | 4558（12.3） | 1505（9.8） | 1752 (12.5) | 565（15.8） | 403（17.4） | 333（20.1） | ＜0.001 |
| Previous PCI or CABG | 2663（4.8） | 1147（4.2） | 1036 （5.3） | 247（5.1） | 153（5.7） | 80（5.3） | ＜0.001 | 5081（13.8） | 1837（12.0） | 2074 （14.8） | 557（15.6） | 355（15.3） | 258（15.6） | ＜0.001 |
| Family history of CAD | 1433（2.6） | 820（3.0） | 472 (2.4) | 74（1.5） | 40（1.5） | 27（1.8） | ＜0.001 | 1048（2.8） | 534（3.5） | 353 (2.5) | 85（2.4） | 38（1.6） | 38（2.3） | ＜0.001 |
| Previous AF | 791（1.4） | 169（0.6） | 356 (1.8) | 134（2.8） | 82（3.1） | 50（3.3） | ＜0.001 | 1438（3.9） | 240（1.6） | 607(4.3) | 242（6.8） | 201（8.7） | 148（8.9） | ＜0.001 |
| Hypertension | 34275（61.7） | 15492 （57.1） | 12441 (64.1) | 3303（68.7） | 1893（71.0） | 1146（75.5） | ＜0.001 | 26821（72.6） | 10185（66.3） | 10432 (74.3) | 2891（80.9） | 1881（81.3） | 1432（86.4） | ＜0.001 |
| Hyperlipidemia | 48532（87.3） | 24099 (88.7) | 16934 (87.2) | 4076（84.8） | 2226（83.5） | 1197（78.8） | ＜0.001 | 30886（83.6） | 13023 (84.8) | 11705 (83.4) | 2960（82.8） | 1898（82.1） | 1300（78.5） | ＜0.001 |
| Diabetes | 14309（25.7） | 6664（24.5） | 4755 (24.5) | 1442（30.0） | 886（33.2） | 562（37.0） | ＜0.001 | 11630（31.5） | 4433（28.9） | 4145 (29.5) | 1251（35.0） | 980（42.4） | 821（49.5） | ＜0.001 |
| Previous stroke | 4485（8.1） | 1531（5.6） | 1804 (9.3) | 573（11.9） | 374（14.0） | 203（13.4） | ＜0.001 | 3902（10.6） | 1109（7.2） | 1544 (11.0) | 555（15.5） | 389（16.8） | 305（18.4） | ＜0.001 |
| Previous heart failure | 497（0.9） | 90 （0.3） | 163 (0.8) | 93（1.9） | 76（2.8） | 75（4.9） | ＜0.001 | 1573（4.3） | 206 （1.3） | 606 (4.3) | 277（7.8） | 236（10.2） | 248（15.0） | ＜0.001 |
| Cigarette smoking | 26797（48.2） | 15203（56.0） | 8495 (43.7) | 1739（36.2） | 906（34.0） | 454（29.9） | ＜0.001 | 392（23.7） | 6856 （44.7） | 4734 (33.7) | 979（27.4） | 576（24.9） | 392（23.7） | ＜0.001 |
| Statins before admission | 6785（12.2） | 3177（11.7） | 2462 (12.7) | 564（11.7） | 356（13.3） | 226（14.9） | ＜0.001 | 9519（25.8） | 3640（23.7） | 3722 (26.5) | 989（27.7） | 687（29.7） | 481（29.0） | ＜0.001 |
| Antiplatelet drugs before admission | 10652（19.2） | 5023（18.5） | 3834 (19.7) | 925（19.2） | 536（20.1） | 334（22.0） | ＜0.001 | 12007（32.5） | 4579（29.8） | 4722 (33.6) | 1262（35.3） | 833（36.0） | 611（36.9） | ＜0.001 |
| β-blocker before admission | 3362（6.0） | 1541（5.7） | 1261（6.5） | 303（6.3） | 135（5.1） | 122（8.0） | ＜0.001 | 5511（14.9） | 2058（13.4） | 2188（15.6） | 573（16.0） | 399（17.3） | 293（17.7） | ＜0.001 |
| ACEI/ARB before admission | 3900（7.0） | 1654（6.1） | 1466（7.5） | 421（8.8） | 226（8.5） | 133（8.8） | ＜0.001 | 5796（15.7） | 2008（13.1） | 2353（16.8） | 725（20.3） | 448（19.4） | 262（15.8） | ＜0.001 |
| Heart failure on admission | 4087（7.4） | 1142（4.2） | 1524 (7.8) | 608（12.6） | 451（16.9） | 362（23.8） | ＜0.001 | 2523（6.8） | 403（2.6） | 925 (6.6) | 438（12.3） | 394（17.0） | 363（21.9） | ＜0.001 |
| Cardiac arrest on admission | 1220（2.2） | 394（1.5） | 454 (2.3) | 164（3.4） | 132（4.9） | 76（5.0） | ＜0.001 | 229（0.6） | 61 （0.4） | 75 (05) | 33（0.9） | 33（1.4） | 27（1.6） | ＜0.001 |
| Cardiogenic shock on admission | 2096（3.8） | 544 （2.0） | 727 (3.7) | 363（7.5） | 278（10.4） | 184（12.1） | ＜0.001 | 2523（6.8） | 403 （2.6） | 925 (6.6) | 438（12.3） | 394（17.0） | 363（21.9） | ＜0.001 |
| SBP on admission (mmHg) | 127.4±23.4 | 27.9±22.0 | 127.7±23.5 | 125.7±25.8 | 124.5±27.5 | 126.6±29.6 | 0.001 | 135.5±22.8 | 134.2±21.4 | 136.0±22.6 | 136.9±23.8 | 136.4±26.2 | 139.3±27.8 | 0.001 |
| DBP on admission (mmHg) | 77.8±14.8 | 79.4±14.3 | 77.1±14.4 | 75.2±15.7 | 73.8±16.2 | 74.8±17.4 | ＜0.001 | 79.0±13.7 | 80.6±13.3 | 78.2±13.4 | 77.8±14.4 | 77.1±14.9 | 76.9±15.8 | ＜0.001 |
| HR on admission (bpm) | 78.1±16.5 | 77.7±15.0 | 77.6±16.6 | 79.5±19.3 | 80.2±20.8 | 81.6±22.0 | ＜0.001 | 76.7±15.7 | 74.9±13.6 | 76.3±15.6 | 78.7±18.0 | 82.4±19.8 | 83.6±19.4 | ＜0.001 |
| Killip class≥3 | 5289 (9.5) | 1365（5.0） | 1945 (10.0) | 850（17.7） | 664（24.9） | 465（30.6） | ＜0.001 | 4367 (11.8) | 936 （6.1） | 1630 (11.6) | 701（19.6） | 546（23.6） | 554（33.4） | ＜0.001 |
| eGFR on admission (ml/min·1.73m^2^) | 88.0 (68.3-99.8) | 101.5（95.8-108.7） | 78.9（70.5-85.2） | 53.62  (49.57-57.04) | 38.66  (35.03-42.01) | 21.81  (14.47-26.27) | ＜0.001 | 88.0 (68.3-99.8) | 99.7（94.8-106.2） | 78.6（69.8-85.0） | 53.44  (49.35-56.72) | 38.60  (34.68-41.94) | 20.62  (13.36-25.68) | ＜0.001 |
| HBA1C，(%)* | 6.1（5.6-7.2） | 6.0 (5.5-7.3) | 6.0 (5.6-7.0) | 6.1 (5.7-7.2) | 6.3 (5.7-7.5) | 6.4(5.8-7.9) | ＜0.001 | 6.1（5.6-7.2） | 6.1 (5.6-7.3) | 6.1 (5.7-7.1) | 6.2 (5.7-7.4) | 6.4(5.7-7.7) | 6.4(5.7-7.5) | ＜0.001 |
| HB on admission (g/L) | 137.7±20.3 | 142.3±18.6 | 136.0±19.4 | 131.4±20.7 | 126.7±21.8 | 117.8±25.6 | ＜0.001 | 133.5±20.7 | 139.2±18.6 | 133.5±19.0 | 127.3±20.2 | 122.5±22.1 | 109.5±24.9 | ＜0.001 |
| TC (mmol/L) | 4.48±1.22 | 4.56±1.23 | 4.43±1.19 | 4.39±1.23 | 4.32±1.24 | 4.26±1.33 | ＜0.001 | 4.36±1.25 | 4.42±1.24 | 4.32±1.23 | 4.33±1.27 | 4.32±1.35 | 4.21±1.38 | ＜0.001 |
| TG (mmol/L) | 1.44 (1.02-2.14) | 1.51（1.05-2.25） | 1.35（0.96-1.99） | 1.32（0.94-1.93） | 1.35  (0.93-2.03) | 1.39  (1.01-2.05) | ＜0.001 | 1.44 (1.02-2.14) | 1.55（1.10-2.30） | 1.42（1.01-2.08） | 1.46（1.01-2.14） | 1.41  (0.98-2.14) | 1.47  (1.02-2.17) | ＜0.001 |
| LDL-C (mmol/L) | 2.69  (2.08-3.35) | 2.76  (2.19-3.40) | 2.69  (2.12-3.31) | 2.65 (2.05-3.28) | 2.56  (2.02-3.22) | 2.44  (1.86-3.14) | ＜0.001 | 2.69  (2.08-3.35) | 2.61 (2.01-3.27) | 2.54  (1.96-3.21) | 2.54 (1.91-3.22) | 2.54  (1.88-3.25) | 2.40  (1.78-3.13) | ＜0.001 |
| HDL-C (mmol/L) | 1.01  (0.85-1.22) | 1.03  (0.87-1.24) | 1.05  (0.87-1.26) | 1.05 (0.87-1.27) | 1.04  (0.85-1.27) | 1.01（0.82-1.24） | ＜0.001 | 1.01  (0.85-1.22) | 1.03  (0.86-1.24) | 1.04  (0.87-1.25) | 1.03  (0.85-1.26) | 1.02  (0.84-1.27) | 0.98（0.78-1.23） | ＜0.001 |
| Undergoing CA | 45512 (81.9) | 23487(86.5) | 15795 (81.3) | 3572 (74.3) | 1788（67.0） | 870 (57.3) | ＜0.001 | 24850(67.3) | 11556(75.3) | 9445 (67.3) | 2068 (57.9) | 1151（49.8） | 630 (38.0) | ＜0.001 |
| Undergoing PCI | 43566（78.4） | 22459（82.7） | 15148 (78.0) | 3422（71.2） | 1698（63.7） | 839（55.2） | ＜0.001 | 20286（54.9） | 9567 （62.3） | 7651 (54.5) | 1648（46.1） | 913（39.5） | 507（30.6） | ＜0.001 |
| Coronary artery lesions |  |  |  |  |  |  |  |  |  |  |  |  |  |  |
| One-vessel lesion | 37969(68.3) | 18842（69.4） | 12951 (66.7) | 3236 (67.3) | 1558 (48.7) | 766 (46.1) | ＜0.001 | 42416(57.1) | 22254（60.9） | 14695 (54.8) | 3143 (51.9) | 1558 (48.7) | 766 (46.1) | ＜0.001 |
| multi-vessel lesions | 15796(34.7) | 7581（32.3） | 5754 (36.4) | 1384 (38.7) | 729 (40.8) | 348 (40.0) | ＜0.001 | 12598(50.7) | 5438（47.1） | 4950 (52.4) | 1146 (55.4) | 669 (58.1) | 395 (62.7) | ＜0.001 |
| Left main vessel lesions | 1809（4.0） | 731（3.1） | 720 (4.6) | 189（5.3） | 116（6.5） | 53（6.1） | ＜0.001 | 2070（8.3） | 740（6.4） | 861 (9.1) | 221（10.7） | 141（12.3） | 107（17.0） | ＜0.001 |

Values are mean±SD, median(interquartile range, IQ), or n (%).

CKD：chronic kidney disease；eGFR: estimated glomerular filtration rate；ACS：acute coronary syndrome；STEMI：ST-elevation myocardial infarction；NSTEMI：Non-ST elevation myocardial infarction；UA：Unstable angina pectoris；PCI：percutaneous coronary intervention；CABG：coronary artery bypass grafting;；SBP: systolic blood pressure; DBP: diastolic blood pressure; HR: heart rate; ACEI：Angiotensin Converting Enzyme Inhibitors；ARB：Angiotensin receptor antagonist; FBG: fasting blood glucose; HBA1C: glycated hemoglobin; HB:Hemoglobin; TG：triglyceride；TC：total cholesterol；LDL-C：low-density lipoprotein cholesterol；HDL：high-density lipoprotein cholesterol.

HBA1C was not available for 52196(56.4%) patients

**Table S4** In-hospital outcomes of STEMI and NSTE-ACS patients

| STEMI | | | | | | | | NSTE-ACS | | | | | | |
| --- | --- | --- | --- | --- | --- | --- | --- | --- | --- | --- | --- | --- | --- | --- |
|  | Total  （n=55574 ） | eGFR≥90ml/min·1.73m^2^  （n=27154,  48.86%） | eGFR60-89 ml/min·1.73m^2^  （n=19425,  34.95%） | eGFR45-59 ml/min·1.73m^2^  （n=4809,  8.65%） | eGFR30-44 ml/min·1.73m^2^  （n=2667,  4.80%） | eGFR＜  30 ml/min·1.73m^2^  （n=1519,  2.73%） | P value | Total  （n=36933 ） | eGFR≥90ml/min·1.73m^2^  （n=15352,  41.57%） | eGFR60-89 ml/min·1.73m^2^  （n=14037,  38.00%） | eGFR45-59 ml/min·1.73m^2^  （n=3574,  9.68%） | eGFR30-44 ml/min·1.73m^2^  （n=2313,  6.26%） | eGFR＜  30 ml/min·1.73m^2^  （n=1657,  4.49%） | P value |

| death | 1185（2.1） | | 211（0.8） | | 389（2.0） | | 230（4.8） | 189（7.1） | | 166（10.9） | | ＜0.001 | 433（1.2） | 50（0.3） | 119（0.8） | 80（2.2） | 81（3.5） | | 103（6.2） | ＜0.001 |
| --- | --- | --- | --- | --- | --- | --- | --- | --- | --- | --- | --- | --- | --- | --- | --- | --- | --- | --- | --- | --- |
| Cardiogenic shock | 1942（3.5） | | 485（1.8） | | 665（3.4） | | 322（6.7） | 273（10.2） | | 197（13.0） | | ＜0.001 | 466（1.3） | 74（0.5） | 143（1.0） | 78（2.2） | 87（3.8） | | 84（5.1） | ＜0.001 |
| Heart failure | 4906（8.8） | | 1429（5.3） | | 1864（9.6） | | 716（14.9） | 509（19.1） | | 388（25.5） | | ＜0.001 | 2529（6.8） | 445（2.9） | 906（6.5） | 424（11.9） | 404（17.5） | | 350（21.1） | ＜0.001 |
| Cardiac arrest | 1131（2.0） | | 261（1.0） | | 386（2.0） | | 195（4.1） | 162（6.1） | | 127（8.4） | | ＜0.001 | 328（0.9） | 48（0.3） | 88（0.6） | 60（1.7） | 63（2.7） | | 69（4.2） | ＜0.001 |
|  |  |  | |  | |  | |  |  | |  | |  |  |  |  |  |  |  |  |

Values are median (interquartile range, IQ), or n (%).

ACS：acute coronary syndrome；STEMI：ST-elevation myocardial infarction
